# Draft Genome Sequence of the Mannitol-Producing Strain *Lactobacillus mucosae* CRL573

**DOI:** 10.1128/genomeA.01292-14

**Published:** 2014-12-11

**Authors:** Juliana Bleckwedel, Lucrecia C. Terán, Julieta Bonacina, Lucila Saavedra, Fernanda Mozzi, Raúl R. Raya

**Affiliations:** Centro de Referencia para Lactobacilos (CERELA) – CONICET, San Miguel de Tucumán, Argentina

## Abstract

*Lactobacillus mucosae* CRL573, isolated from child fecal samples, efficiently converts fructose and/or sucrose into the low-calorie sugar mannitol when cultured in modified MRS medium at pH 5.0. Also, the strain is capable of producing bacteriocin. The draft genome sequence of this strain with potential industrial applications is presented here.

## GENOME ANNOUNCEMENT

*Lactobacillus mucosae* is an obligate heterofermentative lactic acid bacterium phylogenetically related to *Lactobacillus reuteri*, *Lactobacillus fermentum*, and *Lactobacillus pontis* (1). *L. mucosae* CRL573 (CERELA Culture Collection), isolated from child fecal samples in Tucumán, Argentina, was originally identified as *L. fermentum* and later reclassified through 16S rRNA gene sequencing (LX03_06360) as *L. mucosae* (our unpublished data). This strain efficiently produces mannitol from fructose and sucrose, synthesizing 312 mM mannitol in modified MRS medium at pH 5.0 (2). Also, this strain produces an uncharacterized bacteriocin.

The genome of *L. mucosae* CRL573 was sequenced (50-fold coverage) using a whole-genome shotgun (WGS) strategy with an Ion Torrent personal genome machine (Life Technologies). All reads were assembled into 38 contigs using NGen (DNAStar). The functional annotation was performed by the NCBI Prokaryotic Genomes Annotation Pipeline (PGAAP) (http://www.ncbi.nlm.nih.gov/genome/annotation_prok/). tRNAs and rRNAs were identified by tRNAscan-SE (3).

The genome size consists of 2,257,701 bp, with an overall G+C content of 46.6%. Also, 2,355 predicted open reading frames, 23 rRNAs, and 134 tRNAs were detected. Two hundred ninety-three subsystems were found using RAST (4, 5). Additionally, two intact and four incomplete prophages (PHAge Search Tool [PHAST] [6]), a clustered regularly interspaced short palindromic repeat (CRISPR) element (CRISPRFinder tool [http://crispr.u-psud.fr] [7]), and two potential enterolysin A genes (bacteriocin class III; >10 kDa) (Bagel 3 tools [http://bagel.molgenrug.nl/index.php/bagel3]) were found. The presence of a mucus-binding protein (*mub*) gene is a common characteristic in *L. mucosae* species (i.e., strains S5, S14 and S15, S17 and S32, and LM1) (1, 8); however, a *mub* pseudogene in the CRL573 genome was observed, suggesting that this bacterium lacks the ability to adhere to pig mucus *in vitro* (8).

As mentioned above, *L. mucosae* CRL573 is an efficient mannitol producer, a polyol with multiple industrial applications mainly used as natural sweetener in the food industry due to its low-caloric, low-glycemic, and anticariogenic properties. A mannitol-dehydrogenase (*mdh*) gene (LX03_09970), a homolog to the *mdh* gene of *L. reuteri* CRL1101, was found in CRL573, although it was disrupted by a single stop codon. Resequencing of this region confirmed the *mdh* pseudogene. The mannitol-dehydrogenase (MDH) protein is a member of the medium-chain dehydrogenase/reductase (MDR)/zinc-dependent alcohol dehydrogenase-like family (9). Members of the MDR group, including alcohol dehydrogenase (ADH), sorbitol dehydrogenase, and ketose reductase, exhibit broad activities. The zinc-dependent alcohol dehydrogenases (ADHs) catalyze the NADPH-dependent interconversion of alcohols into their corresponding aldehydes or ketones. The disrupted MDH protein encoded by the CRL573 genome consists of the N-terminal catalytic domain and loses the C-terminal NADP binding-Rossmann fold domain of the MDR proteins. Two other genes present in the CRL573 genome (LX03_02465 and LX03_11755) have the same motif as the MDR group.

A comparison between the *L. mucosae* CRL573 and LM1 genomes shows an identity of >63%. The genome size, subsystem coverage, subsystem number, and number of sequenced contigs are higher in CRL573 than those in the LM1 strain.

### Nucleotide sequence accession numbers.

This whole-genome shotgun project has been deposited at DDBJ/EMBL/GenBank under the accession no. JROC00000000. The version described in this paper is version JROC01000000.
